# Reconstructing Mammalian Sleep Dynamics with Data Assimilation

**DOI:** 10.1371/journal.pcbi.1002788

**Published:** 2012-11-29

**Authors:** Madineh Sedigh-Sarvestani, Steven J. Schiff, Bruce J. Gluckman

**Affiliations:** 1 Center for Neural Engineering, Department of Engineering Science and Mechanics, The Pennsylvania State University, University Park, Pennsylvania, United States of America; 2 Department of Neurosurgery, The Pennsylvania State University, University Park, Pennsylvania, United States of America; 3 Department of Physics, The Pennsylvania State University, University Park, Pennsylania, United States of America; 4 Department of Bioengineering, The Pennsylvania State University, University Park, Pennsylvania, United States of America; École Normale Supérieure, College de France, CNRS, France

## Abstract

Data assimilation is a valuable tool in the study of any complex system, where measurements are incomplete, uncertain, or both. It enables the user to take advantage of all available information including experimental measurements and short-term model forecasts of a system. Although data assimilation has been used to study other biological systems, the study of the sleep-wake regulatory network has yet to benefit from this toolset. We present a data assimilation framework based on the unscented Kalman filter (UKF) for combining sparse measurements together with a relatively high-dimensional nonlinear computational model to estimate the state of a model of the sleep-wake regulatory system. We demonstrate with simulation studies that a few noisy variables can be used to accurately reconstruct the remaining hidden variables. We introduce a metric for ranking relative partial observability of computational models, within the UKF framework, that allows us to choose the optimal variables for measurement and also provides a methodology for optimizing framework parameters such as UKF covariance inflation. In addition, we demonstrate a parameter estimation method that allows us to track non-stationary model parameters and accommodate slow dynamics not included in the UKF filter model. Finally, we show that we can even use observed discretized sleep-state, which is not one of the model variables, to reconstruct model state and estimate unknown parameters. Sleep is implicated in many neurological disorders from epilepsy to schizophrenia, but simultaneous observation of the many brain components that regulate this behavior is difficult. We anticipate that this data assimilation framework will enable better understanding of the detailed interactions governing sleep and wake behavior and provide for better, more targeted, therapies.

## Author Summary

Mathematical models are developed to better understand interactions between components of a system that together govern the overall behavior. Mathematical models of sleep have helped to elucidate the neuronal cell groups that are involved in promoting sleep and wake behavior and the transitions between them. However, to be able to take full advantage of these models one must be able to estimate the value of all included variables accurately. Data assimilation refers to methods that allow the user to combine noisy measurements of just a few system variables with the mathematical model of that system to estimate all variables, including those originally inaccessible for measurement. Using these techniques we show that we can reconstruct the unmeasured variables and parameters of a mathematical model of the sleep-wake network. These reconstructed estimates can then be used to better understand the underlying neuronal behavior that results in sleep and wake activity. Because sleep is implicated in a wide array of neurological disorders from epilepsy to schizophrenia, we anticipate that this framework will enable better understanding of the link between sleep and the rest of the brain and provide for better, more targeted, therapies.

## Introduction

Great strides have been made in understanding the physiological basis for sleep regulation [1] in terms of the interacting neuronal cell groups and their neurotransmitter mediated interactions. This physiology is now increasingly being embodied into complex mathematical models of sleep dynamics [2]–[6]. But the limits to which these models are either validated or otherwise utilized for insight and prediction is currently limited. Due to physical and technological constraints, simultaneous measurement of the physiology embodied in the models - such as cell group firing rates and neurotransmitter concentrations - is not feasible in freely behaving animals or people. We demonstrate here that such models of the sleep-wake regulatory system can be put into a data assimilation framework that allows for reconstruction and forecasting of unobserved dynamics from limited noisy measurements. We anticipate these tools will help shed light on core brain circuitry implicated in sleep disorders as well as sleep-related neurological disorders such as epilepsy [7], bipolar disorder [8], and generalized anxiety disorder [9].

Data assimilation is an iterative process that couples and synchronizes mathematical models to observed system dynamics with the purpose of estimating both noisy observed and unobserved variables, as well as forecasting the future system state. Data assimilation algorithms for nonlinear systems often employ the ensemble Kalman filters [10]. One such ensemble filter is the unscented Kalman filter (UKF), used in an iterative prediction-correction scheme in which model-generated predictions are corrected to agree with or track experimental observations [11].

The objectives of this article are to demonstrate data assimilation applicability within the context of relatively high-dimensional nonlinear biological models of the sleep-wake regulatory system, and to investigate the observability properties of these models [4], [12]. In the Materials and Methods section, we introduce these models, as well as the basic mathematics of the UKF and parameter estimation algorithms. In the Results section, we demonstrate the use of the UKF to reconstruct data generated from these models. We introduce a reconstruction quantification that allows one to gauge the relative observability of the model variables. We demonstrate how this empirical observability coefficient can be used to optimize UKF parameters such as model covariance inflation, as well as how to select the optimal variables for measurement. We then demonstrate a method for optimizing model parameters for tracking slowly varying dynamics. Finally, we demonstrate that we can use measurements of discretized sleep-state generated from the model, instead of specific model variables, to reconstruct unobserved model dynamics.

## Materials and Methods

Data assimilation is an iterative process that couples and synchronizes mathematical models to observed system dynamics. For illustration of the data assimilation framework and validation of results, we use artificially generated data from the Diniz Behn and Booth (DB) model [4] or its extension by Fleshner, et al. (FBFD model) [12]. We then select a subset of the generated variables, to which we add noise, as our measured data set. This data set, with or without the correct parameters used to generate it, is then passed to the UKF to reconstruct the unobserved states (variables) and forecast future system states. Validation is carried out by quantitatively comparing the reconstructed estimates and parameters with the known original data set. A 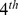 order Runge-Kutta estimate with an integration time of 0.5 seconds is used for all simulations. The MATLAB code to produce each figure in the Results section is available at ModelDB (http://senselab.med.yale.edu/modeldb/default.asp) or can be provided by the authors upon request.

Within this section, we describe both the DB and FBFD models of the sleep-wake regulatory system. We then describe the main features of the UKF and parameter estimation algorithms.

### Physiology of Sleep

Recent advances in single and multi-unit recordings have contributed to the growing knowledge of the mammalian sleep-wake regulatory system. The current prevailing hypothesis includes a *flip-flop* switch that regulates transitions between non rapid-eye-movement sleep (NREM) and wakefulness (Wake) [1]. Gamma-aminobutyric acid (GABA)-ergic ventrolateral preoptic nucleus (VLPO) neurons in the hypothalamus promote NREM. Monoaminergic cell groups in the brainstem, including the noradrenergic locus coeruleus (LC) and the serotonergic dorsal raphe (DR) neurons, promote Wake. Mutual inhibition between these two groups causes each to promote its own activity by inhibiting the other's. McCarley and Hobson [13] described transitions between NREM and rapid eye movement sleep (REM) arising from predator-prey like interactions between cholinergic cell-groups in the brainstem, including the laterodorsal tegmentum (LDT) and pedunculopontine tegmentum (PPT), and the monoaminergic cell-groups in LC and DR. For a more in-depth overview of the literature, including controversial hypotheses for REM regulation, see [14].

More recently, orexin and adenosine have been implicated in further regulation of the sleep-wake system. Orexin producing neurons in the lateral hypothalamus have descending projections to all aforementioned monoamergic and cholinergic cell groups and reinforce arousal, for a review see [15]. Extracellular adenosine has been found to increase during prolonged wakefulness in several cortical and subcortical regions [16], and has been proposed as a homeostatic accumulator of the need to sleep [17].

These dynamics are further modified by the circadian drive [18], regulated by the suprachiasmatic nucleus (SCN) in the hypothalamus, which sets a roughly 24-hour cycle affecting sleep and many other physiological functions. The SCN has indirect projections to the VLPO in the hypothalamus which results in inhibition of sleep during the day [19]. Here day is subjectively defined by species' dependent diurnal behavior, and refers roughly to 12-hour periods consisting mostly of active-wake behavior.

The SCN clock can be modulated by afferent cortical inputs in response to a variety of external cues. Food restriction studies have shown entrainment of the circadian cycle to food availability [20]. Light input from the melonopsin expressing ganglion cells in the retina can also affect the SCN [21]. Retrograde trace studies have shown that a number of central nervous system sites innervate the SCN in the rat [22], though further study is needed to fully elucidate the involved circuitry. For instance, it is well known that lesions of the temporal lobe leading to epileptic seizures also affect the circadian clock [23], [24], but the relevant brain circuitry has yet to be determined.

### Diniz Behn and Booth (DB) Model of Sleep

**Figure 1:**
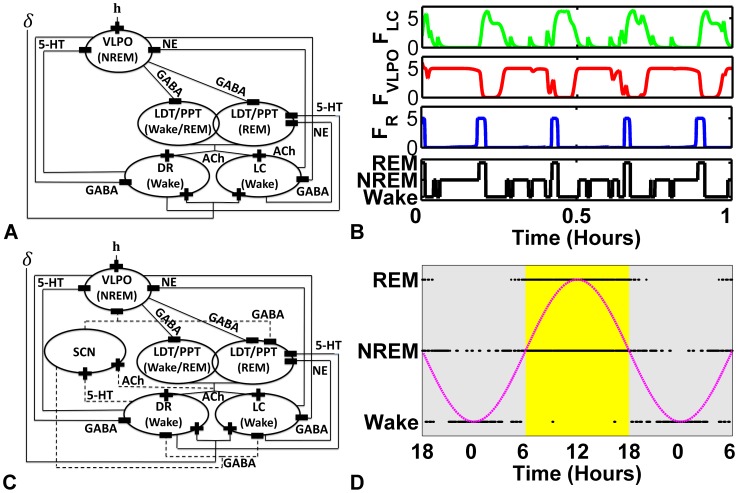
Computational models of the sleep-wake regulatory system and their outputs. A) Diniz Behn and Booth (DB) Model circuit diagram, illustrating the cell groups, their output neurotransmitters, and connections. Inhibitory connections are represented by minus and excitatory connections are represented by plus signs. Locus coeruleus (LC), dorsal raphe (DR), ventrolateral preoptic nucleus (VLPO), laterodorsal/pendunculopontine tegmentum (LDT/PPT), gamma-aminobutyric acid (GABA), seretonin (5-HT), norepinephrine (NE), acetylcholine (ACh), homeostatic sleep-drive (h), non rapid-eye-movement sleep (NREM), rapid-eye-movement sleep (REM). B) Typical output of the DB model for three of the cell group firing rates, plus the scored sleep-state plotted as a hypnogram. C) Fleshner, Booth, Forger and Diniz Behn (FBFD) Model circuit diagram, which expands on the DB model to include circadian modulation by and feedback to the suprachiasmatic nucleus (SCN), and allows for diurnal variations in behavior with light periods dominated by sleep activities and dark periods by periods of extended awake activity. Dashed lines indicate SCN additions to DB model. D) 36 hour hypnogram from the FBFD model, with 24-hour periodic CIRC input input to the SCN superimposed.

The DB model [4], depicted in Fig. 1A, describes interactions among five distinct neuronal populations: two Wake-active groups, LC and DR; two groups in the LDT/PPT, one that is REM-active, denoted R; one active both in Wake and REM, denoted W/R; and one group active during NREM in the VLPO. As illustrated in Fig. 1A, these cell groups communicate through various transmitters: LC transmits norepinephrine (NE), DR transmits serotonin (5-HT), the two groups in the LDT/PPT transmit acetylcholine (ACh), and VLPO transmits GABA. Excitatory thalamic input is modeled by the variable 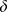 and the brain's homeostatic sleep drive is represented by 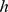. Sample output of this model's sleep-wake cycles, as well as mutual inhibition between Wake and sleep-active regions is shown in Fig. 1B.

Each cell group is described by its firing rate 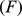 and the concentration 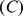 of the neurotransmitter that it releases to post-synaptic populations. Cell group firing rates are a function of their input neurotransmitter concentrations, and evolve according to:
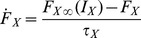
(1)Here 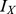 is a weighted sum of neurotransmitter 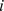 into cell group firing rate 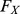, with coupling constants 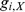;
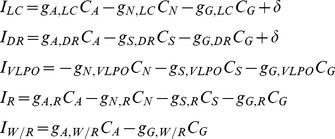
(2)In addition, 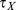 is a first order process time constant. The steady state firing rate, 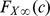, a function of input neurotransmitter 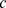, is given by maximum firing rate parameter 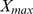, times a sigmoidal function with midpoint 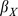 and slope 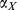:
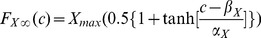
(3)where 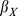 is a constant for all cell groups except VLPO where 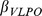 is proportional to the homeostatic sleep drive 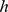. The concentration of neurotransmitter released by each cell group to the post-synaptic space also evolves according to a first order process with time constant 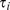 and steady state neurotransmitter concentration 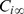 given by:
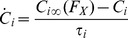
(4)

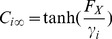
(5)where 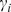 is an adjustable scale parameter.

Because ACh comes from both the R and W/R cell groups, the total ACh concentration in the post-synaptic space is the sum of the ACh concentrations generated individually from these groups. Random excitatory projections from thalamocortical circuits to the Wake-active populations LC and DR are modeled as Poissonian impulses with a rate of 0.003 Hz, which through a leaky integrator form another input concentration denoted 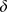 with a decay constant of 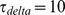 seconds:
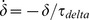
(6)


In addition to firing rate and neurotransmitter concentration variables, the homeostatic drive variable 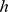 regulates the duration of sleep and wake bouts by changing 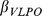, the threshold for firing of the NREM-active VLPO cell group. The accumulation of 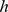 during Wake, and dissipation during sleep, is given by:
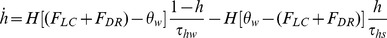
(7)where 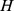 is the Heaviside function, 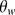 is the threshold parameter for the onset of increase or decrease in 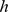, and 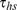 and 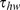 determine the rate of accumulation and dissipation.

Typical output of the DB model is shown in Fig. 1B. The top three traces are the time dynamics of the firing rates for the Wake-active (LC), NREM-active (VLPO), and REM-active (LDT/PPT) cell groups. Note that, following [4] we denote firing rate for the REM-active LDT/PPT cell group as 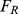. The state of vigilance (SOV), or sleep state, shown as a hypnogram in the fourth trace, is determined by the rank-ordered comparison of these cell group activities, with LDT/PPT activity dominating the definition.

### Fleshner, Booth, Forger and Diniz Behn (FBFD) Model of Sleep

Fleshner, Booth, Forger and Diniz Behn [12] introduced an extension of the DB model that includes the SCN as an additional cell group with GABA as its associated neurotransmitter [12], depicted in Fig. 1C. The firing rate of the SCN cell group follows the same dynamics as the cell groups in the DB model (Eq. 1). The SCN has an inherent 24-hour circadian cycle (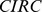), with higher activity during the 12-hour light phase and lower activity during the 12-hour dark phase.

The projections from the sleep-wake network to the SCN provide dynamical feedback that increases the SCN's activity during both Wake and REM and decreases its activity during NREM. The SCN receives 5-HT and ACh synaptic inputs from the core sleep-wake regulatory system through the variable 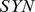. This is modeled by composing 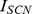 from the sum of 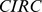 and 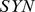. Although the amplitude of 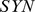 is smaller than that of 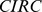, its oscillation time-scale is faster, typically on the order of minutes.
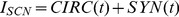
(8)

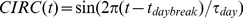
(9)

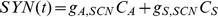
(10)Here 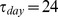 hours. We have shifted the phase of 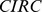 from [12] by adding 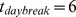 to make the light period (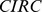 high) start at 6 am. Feed-forward projections of the SCN on the sleep-wake network are mediated through GABAergic transmission, modeled by the additional neurotransmitter concentration 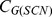, which adds into the dynamics of the LC, DR, VLPO, and R firing rates, modifying Eq. (2) from the DB model to become:
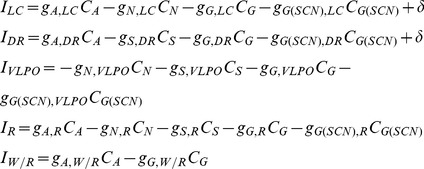
(11)


Typical output of the FBFD model on short time-scales is similar to that of the DB model. But, as is typical for real rats, on diurnal time-scales the typical duration times in different states, as well as cycle times through states, changes. The hypnogram of the output of this model is shown in Fig. 1D for a 36 hour period. Rats are nocturnal. In the model, REM and NREM are primarily observed during the putative light phase, while long periods of Wake are observed during the putative dark phases.

### Unscented Kalman Filter

The Kalman filter estimates the state of a system from noisy, sparsely measured, variables. Kalman's initial filter derivation [25] was for linear systems. The unscented Kalman filter is an ensemble version developed to tolerate nonlinearities without linearization [26].

The details of the UKF algorithm can be found in many standard textbooks [27], [28]. We present here an overview, along with the key equations needed to understand details presented later in this manuscript.

State estimation with the UKF is carried out recursively using a prediction-correction scheme. Each iteration starts with a best estimate 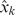 of the current state 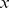 at iteration time 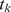. Included is an estimate of the current uncertainty in state 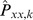. A *prediction* or forecast is then generated by iterating an ensemble of points near 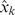, called sigma points, through the nonlinear model dynamics 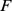. Given a 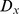 dimensional state space for 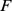, we choose 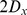 sigma points such that they have covariance 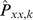 to represent the state uncertainty. We denote the 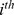 sigma point 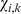 prior to iteration, and 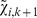 after iteration. The model prediction 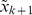 is then the mean of the forward iterated sigma points:
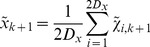
(12)The prediction uncertainty 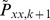 is then the covariance of these points plus an additive covariance inflater matrix 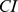.
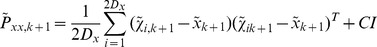
(13)

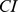
 is nonzero only on the diagonal, and is added to account for underestimates of the forecast error, from the covariance of the sigma points, due to process noise and inadequacies in the filter model [29], [30]. The prediction is then *corrected* to account for a measurement 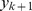 at time 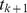. 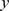 need not contain the same number of variables as 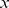. The correction factor weights the observation and prediction according to the Kalman gain 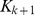:
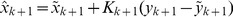
(14)where 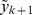 denotes the prediction mean from the estimated sigma points for the observed variables, 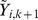 :
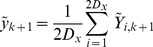
(15)The Kalman gain is formed from the ratio :
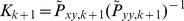
(16)where 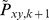 and 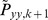 are formed from averages over the sigma points, either in the full dimensional space of 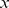 or in the subspace spanned by the measurements:
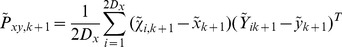
(17)

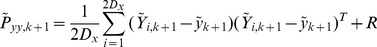
(18)where 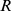 is the measurement uncertainty. The Kalman gain is also used to correct - and ideally collapse - the prediction uncertainty:
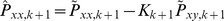
(19)


Within this recursive scheme, the UKF synchronizes model state to measurements and thereby improves the estimate of the experimentally inaccessible variables. *The limit to which this succeeds depends in part on the relative observability of the reconstructed model variables from the measured variables. We discuss below an empirical method for assessing this relative observability*. We note that 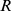, the uncertainty of the measurement process usually can be estimated, using the assumption that measurement noise is normally distributed [31]–[33].

On the other hand, the additive covariance inflation parameter 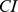 is less clearly defined. Some methods have been proposed to estimate its values under the limited case that its source is an additive process noise [34]–[38]. Within our results - we demonstrate that even with identical system and model dynamics, non-zero 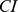 improves tracking, and present a method of optimally choosing the values of 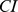 for tracking and prediction.

### Parameter Estimation

One approach for parameter estimation within the UKF framework is to solve the dual problem of estimating parameters and states at the same time, for instance via an augmented state space approach [11]. The alternative approach is to separate state reconstruction from parameter estimation by iteratively alternating between the two [39]. We found that dual estimation did not work well for our high-dimensional sleep-models, likely in part due to the many degrees-of-freedom when neither parameters nor variables were fixed, and especially because in nonlinear systems the sensitivity of the dynamics to particular parameters can be highly dependent on location in state space. We therefore estimate parameters iteratively over windows of length 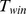 that are longer than a typical sleep-wake cycle of the dynamics.

Within our method, hidden states are first reconstructed with the UKF using a filter model with initial best-guess parameters. The full-state reconstruction over 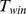 is then used in a parameter estimation step which yields an updated parameter set. This updated parameter set is then passed to the UKF for the next iteration. This process is repeated until the parameter estimate has stabilized.

The parameter estimation step is essentially an application of a multiple shooting method [11], [40]. Within each window, we estimate parameters by creating an average cost-function 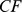 that quantifies the divergence between short model-generated trajectories 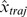 and the UKF-reconstructed trajectories *for the measured variables*. We then minimize this cost-function with respect to the parameter of interest. In order to prevent the model-generated trajectories from diverging too far from the reconstructed ones, we reinitialize 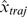 on the reconstructed trajectories at regular intervals 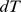:
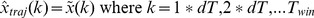
(20)


We then calculate a cost-function averaged over the window 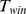 using the divergence between the model-generated trajectories 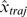 and 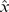:
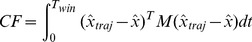
(21)where 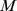 denotes a matrix with non-zero elements on diagonal positions corresponding to measured elements. In order to properly weight the errors for each variable, the non-zero elements of 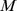 are set to the inverse of the standard deviation of the associated variable. For our current implementation, we perform a minimization with respect to parameters by explicitly computing 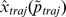 for test parameters 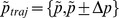 and then choosing the one with the minimum 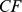. We use a constant value of 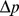, and restrict our parameter update maximally to 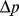 per iteration. Though somewhat computationally intensive, this method yields a stable approach to a local minimum in 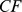. This also limits the resolution to which the parameter can be estimated.

For estimation of non-stationary parameters, we use overlapping windows, with an update period of 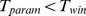. We note that 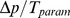 should be greater than the maximal expected rate of change of the parameter of interest, to ensure that parameter dynamics are estimated with reasonable fidelity.

## Results

### Data Assimilation

We can accurately reconstruct unmeasured variables of the DB model of sleep with the UKF framework. To demonstrate this, we generate data from this model, then apply a noisy observation function - the output of which is a noisy subset of the variables - to mimic experimental conditions. We then reconstruct the unobserved variables with the UKF. Finally, we validate this reconstruction by comparing to the original data set.

**Figure 2:**
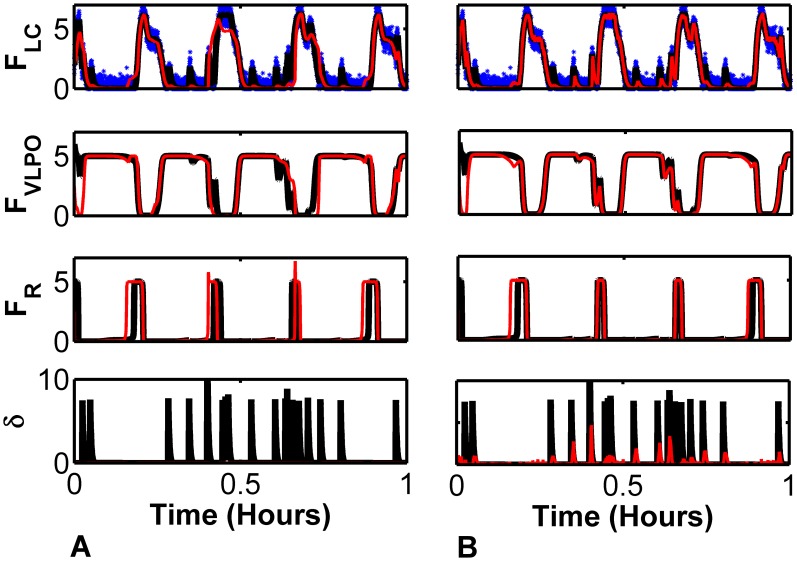
Reconstruction of DB Model Dynamics (A) with default values for covariance inflater 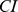, and (B) after optimization of 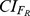 and 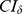. Noisy measurements of 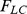 (blue) were passed to the unscented kalman filter (UKF) framework to track and reconstruct all other variables. Shown are the firing rates for the Wake-active (LC), NREM-active (VLPO), and REM-active (LDT/PPT) cell groups, along with thalamic noise 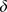. The framework was given the same parameters used to generate the original data. In both A and B the same data was tracked with model initial conditions chosen randomly. After a transient period, reconstructed (red) Wake and NREM dynamics are close to true (black) dynamics. Without 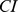 optimization the dynamics of 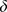 are essentially ignored. After 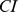 optimization at least some of the stochastic 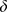 dynamics - those that measurably affect the dynamics of 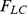 - are reconstructed and reconstruction of REM dynamics is improved.

An example of this procedure is shown in Fig. 2. Long multivariate time series of sleep-wake data were generated from the DB model. The observation function yielded a noisy univariate version of the firing rate of the Wake-active LC region 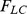. Explicitly we added random, normally-distributed, zero mean noise with variance of 4% that of the variance of 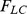 to the true values. We provided the framework the parameters used to generate the original data, and either default values (left panels) or optimized values (right panels) for the covariance inflation parameter 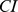. Default values of 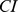 were chosen as 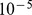 times the typical variance of each variable. Additionally, the initial conditions of the model state, 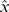, were arbitrarily chosen in each case.

Shown in Fig. 2 are the reconstructed (red) and true (black) values of the NREM-active firing rate variable 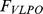, the REM-active firing rate variable 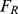 and the stochastic thalamic noise variable 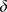. In both cases of tracking, reconstruction of the observed variable 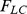 is good. This can be seen from the closeness of the reconstructed traces to both the observation points, shown in blue, and the true values in black. Likewise, the reconstruction of 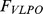 also tracks the true state quite well.

However, for the default values of 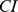, 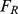 is not reconstructed as well, and 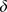 is not reconstructed at all. These errors extend to lower reconstruction fidelity of 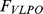 and even of 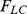. On the other hand, when we use optimized 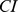 values the reconstruction of 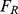 is improved. In addition, much of the thalamic noise input through 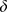 - which is stochastically driven and receives no input from the other variables - is now represented. For these reconstructions, we initialized the model state far from that of the true system state. Therefore, there is a transient period during which reconstruction is poor. In our experience, once the model state comes close to that of the true system, this data assimilation framework keeps the model relatively close to the system state.

#### Quantification of reconstruction fidelity and observability

Visual inspection of the similarity between the reconstructed and true dynamics is only a qualitative result. We therefore use the mean square difference between the reconstructed (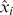) and true (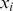) values for each variable to quantify the accuracy of state reconstruction. We normalize this error by the variance of each variable's dynamics to form 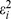, a normalized mean square error for the 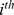 variable :
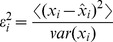
(22)


For perfect reconstruction, 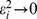. Its maximum depends on the ratio of the full range of the variable to the square root of its variance. For typical variables of the DB model this is of order 3, though for some such as 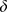 it is 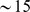. For visualization and regularization purposes, we therefore use the inverse of 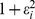, which is bounded between [0,1] as a reconstruction fidelity metric.

The reconstruction fidelity of any particular variable may depend on framework parameters such as integration or reconstruction time step, covariance inflation, as well as qualities that are inherent to the model dynamics such as its observability. Observability is a structural property of a model defined as the ability to recover the model state through the observation of one or more of its outputs [41]. It is well known that not all variables can be used as observables to reconstruct the full dynamics. Nonetheless, information regarding the partial observability of each variable can be used to choose the optimal variable for measurement in the UKF framework.

**Figure 3 pcbi-1002788-g003:**
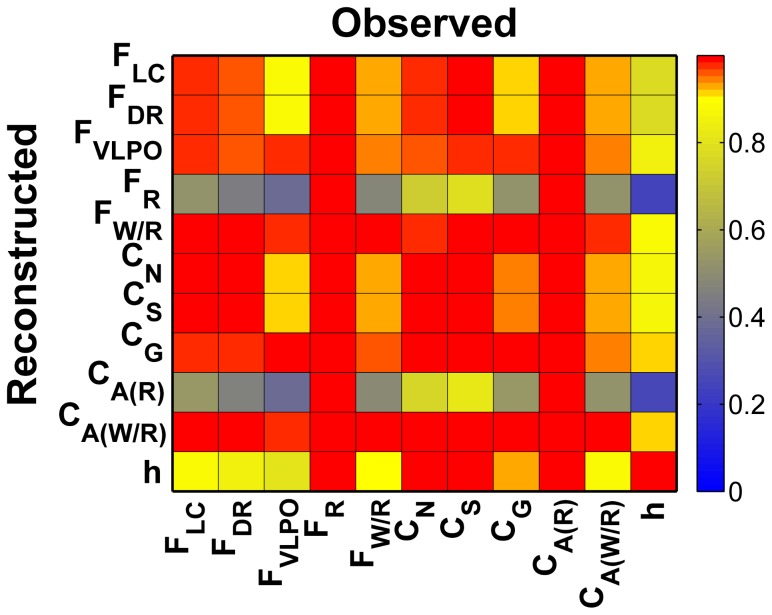
Empirical Observability Coefficient (EOC) matrix. 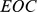
 for the DB model with no thalamic noise and default values of 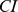. 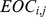 is an empirical measure of how well variable 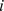 is reconstructed from measurement of variable 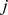. 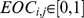 with perfect reconstruction being 1. Here 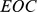 was computed using 12 hours of data. From the 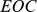 matrix, we observe that 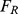 (row) is poorly observed - poorly reconstructed - from most variables, although its measurement (column) yields good reconstruction of almost all other variables.

In Fig. 3, we show the reconstruction fidelity for each variable (down the columns) as a function of observation variable (across rows), in matrix format. We have used constant default values for 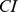, the covariance inflation parameter, relative observation noise 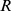 of 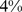 of the variable's variance, and no thalamic noise 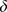. In this color coded plot, red indicates good reconstruction, and blue indicates poor reconstruction.

The diagonal values in this matrix indicate how well a particular variable is reconstructed from itself. Though these tend toward the maximum, they are limited both by the noisy observation as well as the influence of the unobserved variables. Better reconstruction of the full dynamics is indicated by a column that has more red in it. To that extent the best observables for reconstruction are either 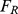, the firing rate of the REM-active group, or its synaptic output 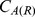. Likewise, the relative reconstruction of a variable from other variables can be gauged by the colors across its row.

Given observation of 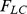, the best reconstructed variable is 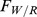, the firing rate of the Wake/REM-active cell group, although several other variables are reconstructed quite well according to high values down the column marked 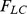. The worst reconstructed variables are 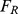 and its synaptic output 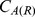. We interpret this to mean that 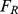 is relatively *less observable* from 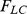 than is 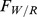. Furthermore, we assert that this is a useful empirical metric for gauging the partial observability of the state space from a measured variable, and name it the *Empirical Observability Coefficient* (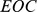):
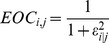
(23)where 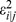 is the normalized reconstruction error for variable 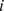 given measurement 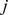. Note for figure labels, we have left off the conditional reference to the measurements.

From the fourth column of the EOC matrix shown in Fig. 3, we observe that 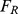 is reconstructed well only if we observe 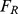 or 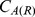, the concentration of ACh that this group transmits to the synaptic space. The poor reconstruction of 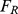 is in part due to symmetries in its dynamics. 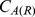 is summed together with 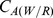, the concentration of ACh transmitted from the Wake/REM-active cell group. As seen in Eq. (2), the total concentration of ACh 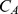, then appears as one of the input transmitters to the Wake-active, REM-active, and Wake/REM-active cell groups. For further discussion of the effect of symmetry on observability and time series reconstruction, see [42].

We did not include dynamics of the thalamic noise variable 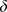 in the model used to generate the 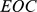 to highlight patterns in the 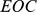 that would be obscured in the presence of this variable.

We note that this empirical observability is related to the *model* used in the UKF framework, not the true dynamics of the system being tracked. In other words, we assess observability in the model. Assuming that the model represents some of the underlying true system dynamics, then those aspects of the true system will also be observable. The computation of the 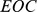 should be done from data generated from the model, not from observed data of the true system.

### EOC Optimization of Covariance Inflation Parameter

We can find optimal framework parameters, such as the UKF covariance inflation parameter matrix 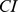, by maximizing 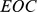 values. Although the matrix 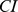 only has nonzero diagonal terms 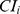, for the full DB model including the thalamic noise output variable 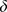, there are 12 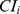
's. So blind simultaneous optimization is inefficient. But we can use the full 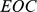, and the ranked partial observability, as a guide to this optimization.

Note from Eq. 13 that 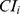 adds to the diagonal elements of the covariance of the sigma points. This inflation has the effect of widening the sigma points on the next iteration step, which results in an increase in the Kalman gain. Larger values for the Kalman gain bias the correction towards the measurements.

Our general rule therefore is that if measurement of a variable 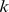 yields poor reconstruction of other variables - i.e. low values of 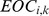 down a column - then we should favor measurement derived values of other variables over model derived ones, and therefore should use *increased* values of 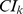. On the other hand, if a variable 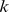 is not reconstructed well from other variables - i.e. low values of 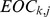 across the row - we should favor model derived values over measurement derived values for this variable by decreasing 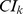.

We iteratively compute the 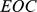, then choose the variable 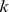 with the lowest scores down a row or column, and change its corresponding 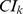 appropriately. We then recompute the 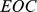 and repeat. This prevents us from optimizing with respect to 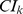
's that have only modest impact on reconstruction fidelity.

There is a finite usable range for 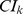. As an inflater for the covariance matrix 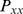, 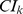 must be greater than or equal to zero. The standard deviation and range of the dynamics of variable 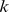 are two natural scales that can be used to define the usable range of 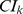. We use the square of the former, multiplied by a proportionality constant, as a default starting value for 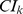. The square of the latter forms the maximum for 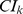.

**Figure 4 pcbi-1002788-g004:**
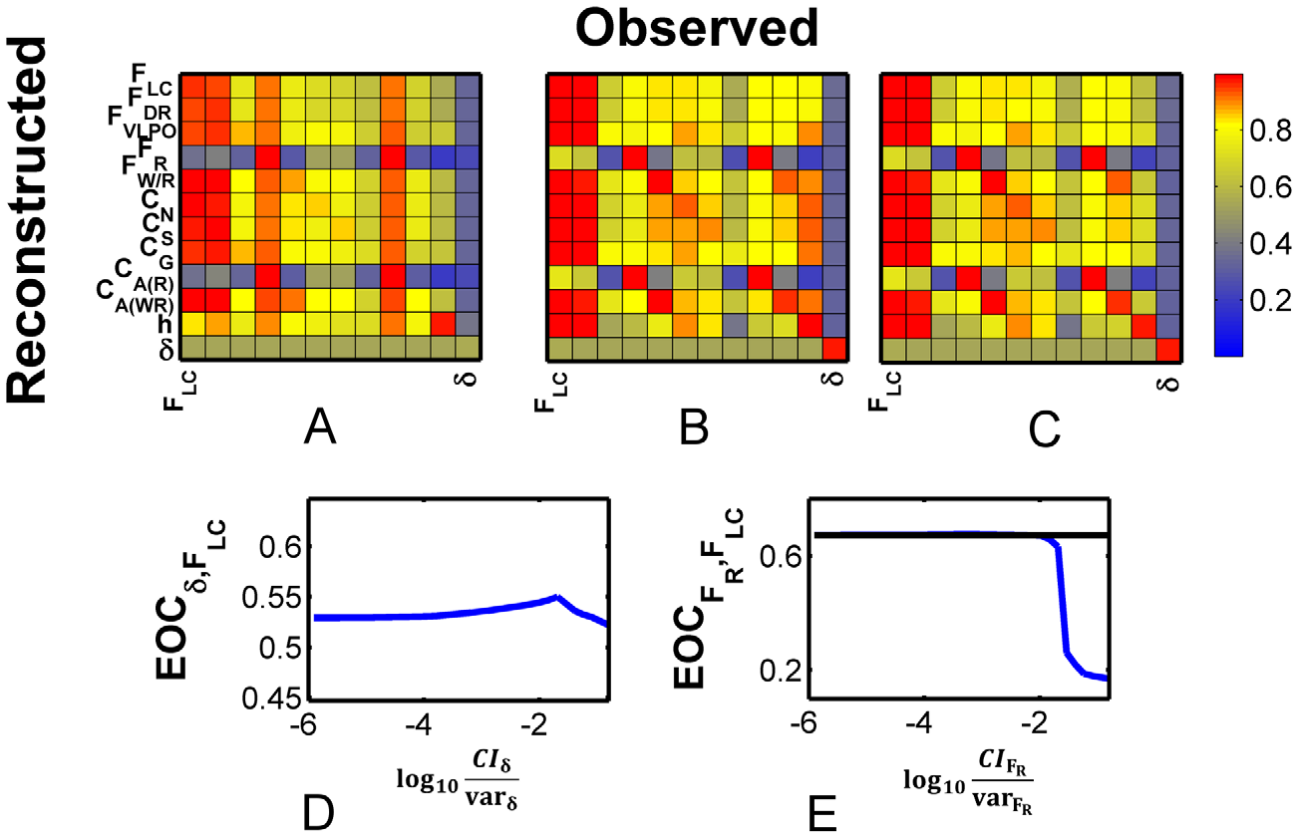
Optimization of Covariance Inflater 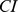. Although the individual EOCs are metrics of reconstruction fidelity, the ranked observability, from the full 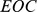 can be used to guide optimization of the covariance inflater 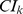: Poorly observed variables across their rows - low 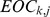 - should have decreased 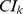. Variables whose measurement yields poor reconstruction columnwise- low 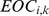 - should have increased 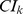. Algorithmically, we iteratively adjust 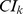 for the variable 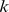 with the overall lowest mean row or column. In A–C are shown the 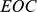 matrix after each optimization iteration for the full DB model with thalamic noise. A) 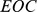 computed with default values for 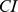, i.e. 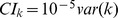. Note that the lowest mean row/column corresponds to the measurement of 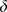, therefore we optimize 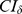 first. B) 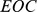 after optimization of 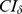. C) 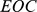 after optimizing 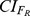. Shown are 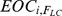 as a function of D) 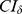 for optimization step between A and B and E) 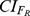 for optimization steps between B and C. Optimal values of 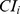 are chosen from the peaks of these plots.

We now demonstrate this algorithm to optimize reconstructions given measurements of 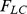, as in Fig. 2B. The full 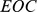 for the DB model - including 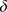 - with default 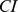 is shown in Fig. 4A. From the far right column, we observed that *no* variables are reconstructed well from measurements of 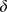. This is understandable, since the dynamics of 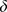 receive no input from any of the other variables. Therefore we start our optimization of 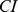 by adjusting 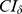, and explicitly expect to increase it.

Shown in Fig. 4D is 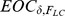 as a function of increasing 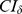. Although only the trace for 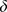 is shown, 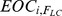 increases for most variables as a function of increasing 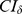. We pick optimal values for 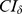 based on the average peak reconstruction of all variables from measurement of 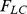, found with a value of 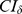 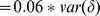. The 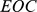 matrix after this first 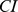 optimization iteration is shown in Fig. 4B.

Notably, although 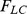 is the variable measured from the real system, its reconstruction improves when 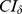 is increased. This effect can be further understood by inspection of Fig. 2. The brief increases in 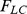 from its low value - interpreted behaviorally as brief awakenings that correlate with spikes in 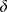 in Fig. 2, are better reconstructed with optimized 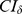. Indeed, the 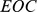 matrix values overall, shown in Fig. 4B, have increased with increasing 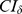.

Now the row/column with the lowest values, on average, corresponds to reconstruction of 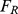. Therefore we expect to need to decrease 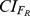 to improve reconstruction. Reconstruction fidelity of 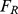 from measurement of 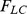, as measured by 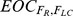 is shown in Fig. 4E as a function of 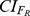. Reconstruction improves with decreasing values over the potential usable range. As shown by the black horizontal line, the best reconstruction is achieved using the minimum value of 0 for 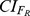, although values of 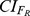 smaller than the default value of 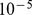 result only in marginal reconstruction improvement. This second optimization step yields only marginal improvement in the overall 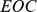 matrix shown in Fig. 4C. In part, this lack of improvement in reconstruction is due to the poor observability of REM dynamics through other variables as apparent from the row marked 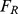 in Fig. 3.

### Empirical Observability and Choice of Measured Variables

We investigated pairings of two or more variables with respect to their relative partial observability. We found that for the DB and FBFD models, the empirical observability of variable 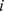 given measurements of variables 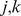 is always at least as good as the individual 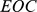: 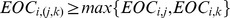. We also observed that good reconstruction of all variables requires some measurement of both Wake and REM dynamics. These states are readily observed from real biological systems from external physiological measurements such as power bands in the EEG, muscle tone, and eye movement. Therefore, for the subsequent computations, we assimilate noisy measurements of both Wake-active 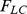 and REM-active 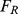 dynamics, and use them to reconstruct the full system state and derive parameter values.

### Parameter Fitting

**Figure 5:**
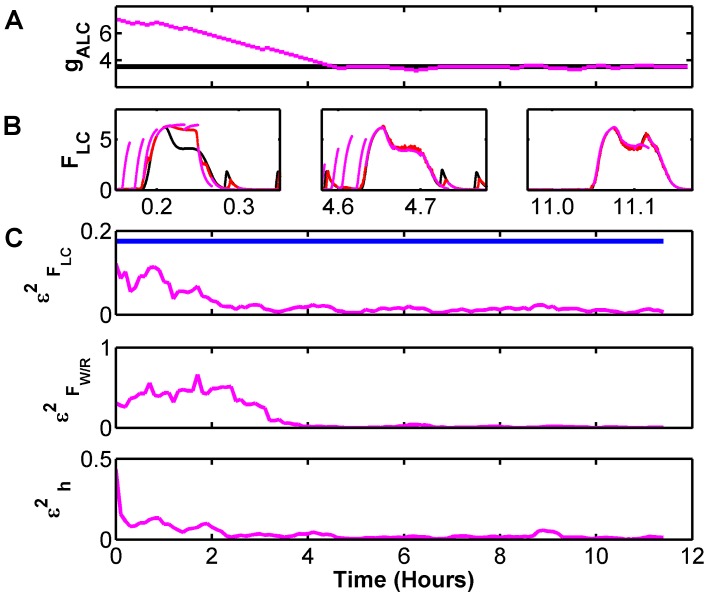
Parameter estimation with multiple shooting method for reconstruction of DB model from measurement of 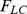 and 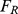 with unknown value for parameter 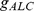. Parameter estimation is performed by minimizing the divergence between the UKF reconstructed dynamics and short model-generated trajectories that originate on the reconstructed trajectories. To sample the full state space, each step of this minimization averages this divergence over time windows longer than the cycle time of the dynamics. Here we use half hour windows, with 80% overlap. A) Convergence of the estimated parameter 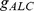 to the true value. B) Trajectories for the short model generated (magenta), reconstructed (red), and true (black) 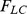 dynamics for different periods of the convergence of 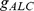. Note that initially, for 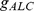 significantly different than the true value, the short trajectories diverge quickly from the reconstructed values, and the reconstructed values of of 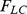 are different from the true values. When 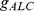 approaches the true value, both short model-generated and reconstructed trajectories approach the true values. C) Reconstruction metric 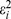 computed for each data assimilation window for three of the variables. As a reference point, the reconstruction metric for the original noisy observation of 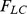 is shown in blue. Note that although the parameter estimation essentially optimizes short model generated forecasts, it has the effect of optimizing hidden variable reconstruction.

As applied here, the UKF framework requires both a model for the dynamics as well as the model's parameters. We have implemented a version of a multiple shooting method [11] for optimizing the choice of parameters. The performance of this method is illustrated in Fig. 5.

For illustration purposes we generated data with fixed parameters and assimilated noisy measurements of 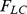 and 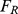 to reconstruct the dynamics. Initially, all model parameters in the UKF were set to the same values used to generate the true data set - except the parameter 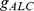 that couples ACh into 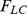 dynamics. To this we supplied an arbitrary initial value.

Parameter estimation is performed by minimizing the distance between the UKF reconstructed traces and short model-generated trajectories that originate on the reconstructed traces. For these computations, we set the length of these short trajectories at 2 minutes. This is long enough that differences in parameters result in measurable divergence between the short computed trajectories and the reconstructed dynamics. Here *measurable* is much larger than the measurement noise, but not so large that the distance between the computed and reconstructed trajectories becomes comparable to the range of the state space.

To sample the full state space, each step of this minimization averages this divergence over time windows longer than the sleep-wake cycle time of the dynamics. As seen in Fig. 5A, our estimation of 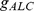 converges to the true value. In Fig. 5B, we plot trajectories for the short model-generated (magenta), reconstructed (red), and true (black) 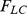 dynamics for different periods of the convergence of 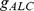. Note that initially, for 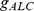 significantly different than the true value, the short trajectories diverge quickly from the reconstructed values, and the reconstructed values of 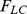 are different from the true ones. When 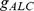 approaches the true value, both the short model-generated and reconstructed trajectories approach the true dynamics.

As coded, the parameter estimation essentially optimizes short model-generated forecasts. To investigate the effect on reconstruction fidelity, we compute the normalized mean square reconstruction error 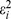 for each variable, averaged over each parameter estimation window. This is shown for variables 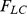, 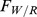, and the homeostatic drive 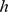. We note that for initial values of this parameter, even reconstruction of the measured variable 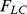 is quite poor - with typical errors 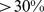 of its standard deviation. As a reference point, the initial measured data - a noisy version of 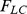 - has a normalized mean squared error 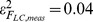, shown as a horizontal blue line. As the estimated parameter converges, 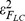 falls well below 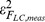, and the reconstruction metric improves for all variables.

### Dynamical Parameter Tracking

We can also estimate parameters which change slowly over time. We demonstrate this by using a slightly modified DB model, which lacks any circadian dynamics, to reconstruct dynamics observed from the expanded FBFD model which specifically includes SCN driven circadian oscillations. We use this modified DB model to assimilate noisy measurements of 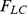 and 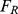 from the full FBFD model, and use it within the multiple shooting method to estimate the value of 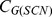.

**Figure 6:**
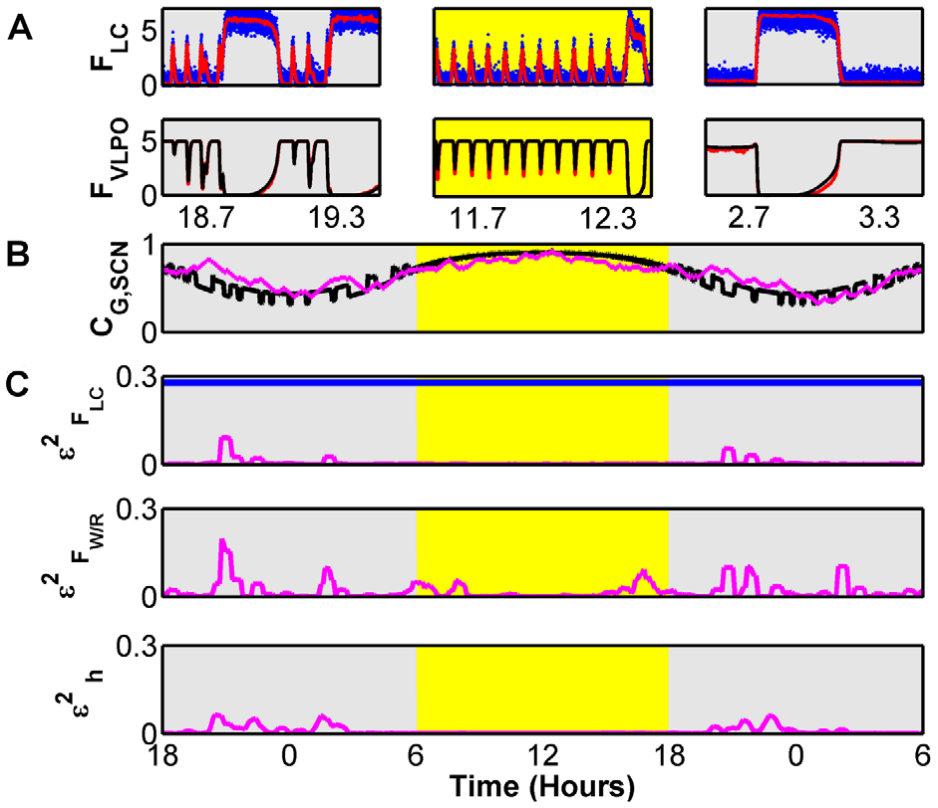
Parameter Tracking to accommodate circadian dynamics. Noisy measurements of 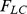 and 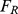 from the full FBFD model were assimilated with a version of the DB model that represented input from the SCN as a quasi-static parameter 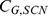 whose value was estimated and tracked in 80% overlapping half hour windows. SCN activity imposes circadian and light-driven dynamics that modulate sleep-wake cycles and prevalence of either sleep or wake activity. A) Short excerpts of reconstructed dynamics for various phases of the circadian cycle. B) Estimated (magenta) and true (black) value of the tracked parameter 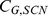. Note that the tracked value is an estimate, with inherent smoothing on the time scale of a half hour, and therefore does not reconstruct all of the detailed dynamics of the true value which oscillates due to the interplay between the core sleep-wake regulatory cell groups and the SCN. C) Normalized reconstruction error for various variables. As a reference, the reconstruction error for the noisy 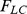 measurement is shown in blue. The reconstruction of unobserved variables 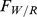 and homeostatic sleep drive 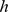 is quite good as indicated by small 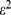 values.

An example of the output is shown in Fig. 6 for a 1.5 day period. We have skipped the initial 12 hours which includes a transient period of convergence of both the filter and the parameter estimate. The effect of the SCN is to modulate the overall sleep cycles, with frequent sleep periods that include REM in the light period and dominant, longer Wake periods in the dark period. Short example time series for 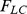 and 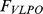 are shown in the panels in Fig. 6A for different phases of the circadian cycle. The filter model in the UKF, also used in parameter estimation, is missing these SCN associated variables and the fast feedback oscillations resulting from their interaction with the sleep network. However, we replace the input contribution of the SCN's feed forward GABAergic projections on to the sleep network to a single quasi-static parameter 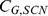 that gets added to other neurotransmitter variables in Eq. (11). We then estimate this parameter which represents the presumed SCN drive.

The estimated value for 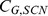 (magenta) is shown in Fig. 6B, along with the true input from SCN in the generating model (black). Though the reconstructed parameter is an estimate with inherent averaging over half-hour periods, and therefore does not reproduce the fast dynamics of the real SCN input, it tracks the mean value quite well. In addition, it yields good reconstruction of the model variables. Examples of the normalized reconstruction error, averaged over the fitting windows, are shown in Fig. 6C for sample variables. Here again, as a reference point, we plot the mean squared error for the noisy measurement of 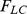 (blue line) in the top panel of Fig. 6C. Note that even reconstruction of the homeostatic sleep drive 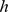, which has no direct coupling to the observed variables, is quite good over most of the day.

### Reconstruction from the Hypnogram

So far, we have implemented the data assimilation framework using measurements that amount to noisy versions of the true variables. In real applications, when one uses observations from real systems, the actual system measurements might only remotely resemble variables in the tracking model. But even in this case, data assimilation methods can still be used. To this end, we demonstrate that we can use measurements of state-of-vigilance (SOV) generated from the model and illustrated in Fig. 1B, to reconstruct the unobserved model dynamics with reasonable fidelity.

**Figure 7:**
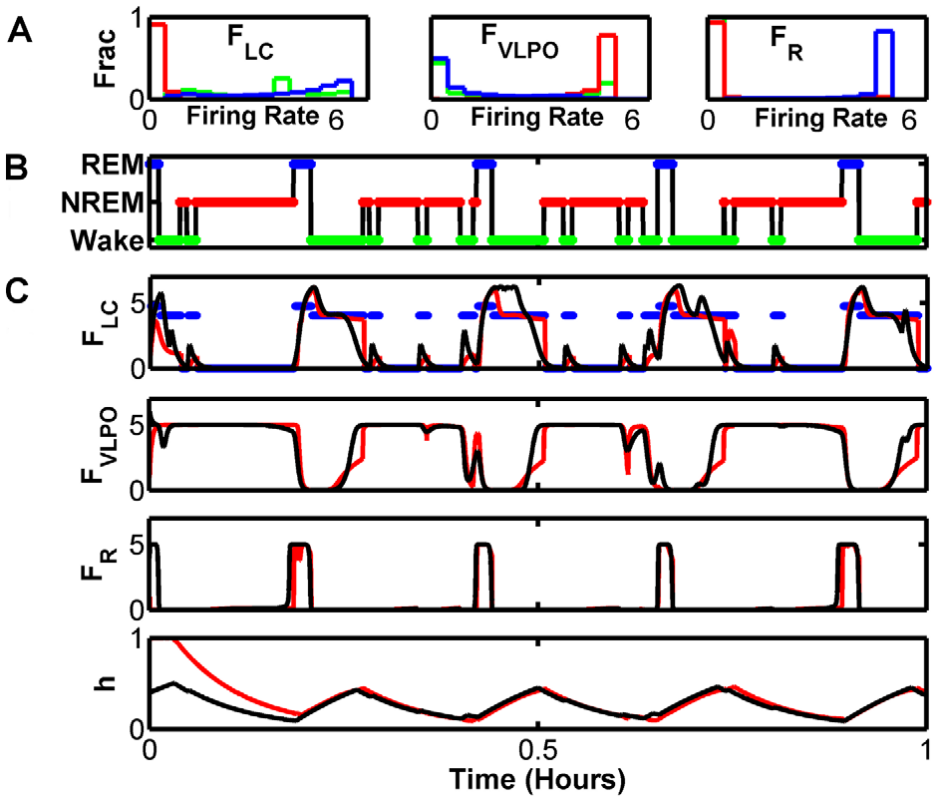
Reconstruction of DB dynamics from measured hypnogram. SOV is used along with an inferred observation function to translate an observed hypnogram into state conditioned observations for 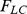, 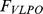, and 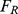, and their variances. We use the UKF to reconstruct the full variable state space from these observations. A) Probability distributions of firing rates for 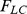, 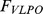 and 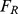 during Wake (black), NREM (red), and REM (blue). These firing rates were generated from the filter-model. B) Hypnogram of observed SOV for a 1 hour time series, with colors to match (A). C) Reconstructed (red) and true (black) traces for 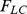, 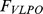, 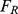, and 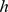. The inferred observation for 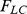 is also shown (blue). After a transient period, the reconstruction converges to the true value, even for the homeostatic drive variable 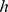 which was not observed. However, details of the dynamics that are not accounted for by the state-of-vigilance (SOV) such as brief awakenings and transitions into and out of NREM are not reconstructed well.

The method we have implemented is illustrated in Fig. 7. We sleep-score the model-generated data, also used in Figs. 2–5, by assigning an SOV to each point as a function of time. The SOV is determined based on relative values of 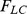, 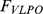, and 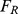. We then take the filter model, and generate example data, which we also sleep-score. From this scored filter-model data, we compute the probability distribution functions (pdf) for the variables 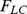, 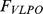, and 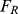 conditioned on SOV. These are illustrated in Fig. 7A. Note that these state-dependent distributions are highly skewed, and have small variance around the mean.

The observation function from the measured values - here SOV as a function of time shown in Fig. 7B - must provide values and error estimates of variables in the filter model to the UKF. To translate the observed SOV to inputs to the UKF, we use the state-conditioned medians from the above-generated pdfs, and then use the state-conditioned standard deviations as the measurement uncertainties. In this way, we use observations of SOV to infer observations of the model variables. We then use these observations, shown for 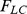 in blue in Fig. 7C, as inputs to the UKF. Note that in this case, the measurement noise estimates are time dependent. After a short transient convergence time, the reconstructed dynamics converge close to the true dynamics. However, certain details such as brief awakenings and transitions into NREM are not reconstructed well.

**Figure 8:**
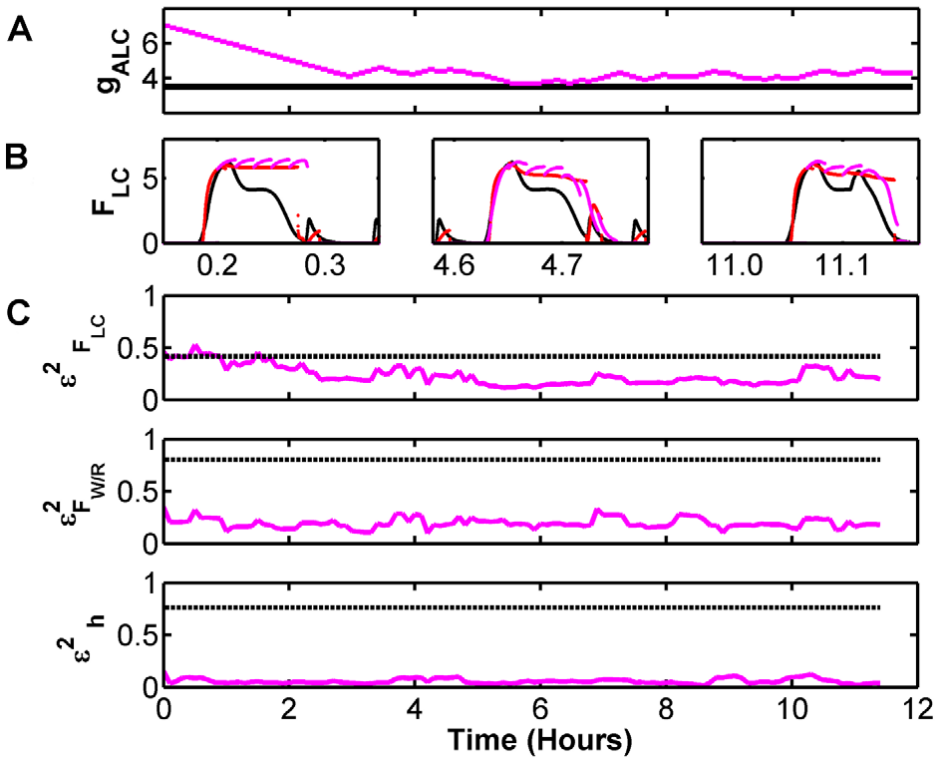
Parameter estimation from observed hypnogram for reconstruction of DB model from inferred measurement of 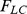, 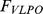, and 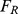 with unknown value for parameter 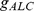. A) Convergence of the estimated parameter(magenta) to the true value (black). B) Trajectories for the short model-generated (magenta), reconstructed (red), and true (black) 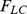 dynamics for different periods of the convergence of 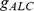. C) Reconstruction metric 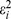 computed for each data assimilation window for three of the variables. Horizontal dashed lines correspond to 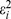 computed from the state-conditioned discrete map used to translate the SOV to model space. Note that once the parameter is optimized, the UKF reconstruction far outperforms the observation map.

We can likewise apply all the other tools described here to assimilation of SOV data through this inferred observation function. Shown in Fig. 8 is the same parameter estimation procedure as shown in Fig. 5, with the same initial conditions for unknown parameter 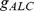. Although the convergence is not as good as with direct observation of 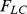, the estimated parameter does approach the parameter used to generate the data. The reconstruction error in 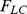 decreases as the parameter approaches its correct value, however neither converge all the way. This can be understood because the UKF attempts to constrain the observed variables to the median values mapped from the SOV. Likewise, the parameter estimation algorithm attempts to minimize the error between model forecasts and reconstructions for the observed variables.

As a supplemental performance metric, we also consider the reconstruction error if we simply use the median observation map for all variables as our reconstruction. These are plotted as horizontal dashed lines for each variable in Fig. 8C. The UKF reconstruction error for the observed variable 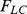 improves beyond this reference point as parameter estimation improves. In contrast, the UKF reconstruction errors for unobserved variables such as 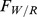 and 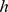 are overall far better.

## Discussion

Data assimilation is a valuable tool in the study of any complex system, where measurements are incomplete, uncertain, or both. It enables the user to take advantage of all available information including experimental measurements and short-term model forecasts of a system. Since the introduction of the UKF to neuronal dynamics by Voss et al. in 2004 [11], a few investigators have applied these methods to the study of biological systems [43]–[48]. Other data assimilation techniques have also been successfully applied to study neuronal dynamics [49]. Nevertheless, the sleep modeling community has yet to utilize these resources. Several important advantages of data assimilation in sleep modeling are : 1) access to unmeasured variables to create a more complete estimate of model state 2) subject-specific parameter estimation even when the parameter is associated with an unobserved variable 3) allowance for uncertainty in model structure or measurements and 4) prediction of future dynamics.

Not all variables perform equally in reconstructing the state space. In biological experiments utilizing data assimilation it would be beneficial to have some insight into the relative performance of each variable so that we can choose the best one or ones for measurement. A natural metric to guide this choice is the observability of each variable. Letellier et al. showed in [50] that observability and ability to synchronize are related. Since the UKF is basically a synchronization scheme, it follows that reconstruction-performance by any variable should be a function of its observability. Thus we propose using observability based metrics in the study of partially observed biological systems.

Analytical methods to determine observability for nonlinear systems are mathematically rigorous, require rational models, and generally do not produce graded values for partial observability. Letellier et al. [51], [52] proposed a simple algebraic solution to rank all variables of a system according to their relative partial observability. Although their approach works well for low-dimensional systems, we found it problematic for our high-dimensional sparsely connected system, where many variables are directly coupled to just one or two other variables, and where the coupling is effectively on only in highly localized regions of state space.

Inspired by their work, we developed an empirical metric, the 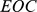, to rank the partial observability of each variable based on reconstructed error. The 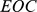 can be used to select the optimal observed variable to obtain the best estimate of a particular unobserved variable. The absolute optimal observed variable receives as input to its dynamics unambiguous invertible information about the state of the unobserved variable. Here invertible implies a one-to-one (bijectvie) relationship between the unobserved and observed variables. In complex networks, this observability is modulated by the number and relative weights of additional unobserved variables in the system that couple into the dynamics of the observed variable [50]–[52].

Because the 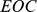 is a measure of reconstruction fidelity, we demonstrate that the reconstruction framework parameters can be optimized by improving it. Importantly, we described an intuitive approach to use the 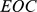 to optimize the covariance inflation parameters 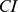. Although some analytical methods have been proposed for this task in nonlinear systems [34]–[38], we are unaware of an observability-based metric for covariance inflation optimization.

Correct parameter estimates aid the prediction of future dynamics and model selection and verification and can provide useful biological information. A common method for parameter estimation in nonlinear models utilizes a feedback-synchronization scheme, developed by [53] and extended by [54]–[58] and many others. Within such a scheme, two identical - except for unknown parameters - systems are unidirectionally coupled, and are continuously synchronized through error feedback. The parameters of the responder are allowed to vary - often using a gradient-decent approach - to minimize a cost function based on driver-responder distance. Although these methods have been shown to work well for systems with smooth variables we found that the sharp transitions in our firing rates, and the highly variable sensitivity of the dynamics to particular parameters as a function of position in state space, resulted in unstable and inaccurate parameter estimates.

We therefore adopted a multiple shooting parameter estimation method [11], [40] that estimates divergence of short model forecasts from the UKF reconstructed trajectories over time windows long enough to explore the state space. This estimation step involves the minimization of a least-squared error, and can therefore be cast as a maximum-likelihood step. This is done in an iterative fashion to update parameter estimates by minimizing divergence of trajectories reconstructed using previous parameter estimates. Therefore this method becomes an expectation-maximization method, with all the associated global optimization implications [59], [60].

Estimation of one or more parameters with any parameter estimation method will be inherently limited by the identifiability of the state space. Identifiability is a structural property of a model defined as the ability to identify a unique set of parameter values given error-free observations of the dynamics [61]. A comparable experimental or empirical version of identifiability has also been discussed by[62]–[65]. If some parameters are not structurally identifiable no parameter estimation method will prevail. Our experience and expectation is that the multiple shooting method will converge reasonably for combinations of identifiable parameters, but the convergence time increases with the number of parameters.

A key advantage of using the UKF for state reconstruction is allowance for uncertainties in the model and/or measurements. As noted, the Kalman filter is an iterative prediction-correction scheme. By altering the elements of the covariance inflation parameters 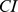 and measurement uncertainty 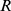, we can guide the Kalman filter to favor either the observations or model predictions. Higher values of 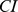 downgrade the model-based forecasts during the correction step. We utilized this when developing the method for optimizing choice of 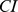 values based on the 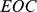. For those variables that are poorly observed from others, we more heavily weight prediction over measurement; for those that yield poor reconstruction of other variables, we more heavily weight measurements.

Furthermore, as we showed in Fig. 6, inadequate models - which omit the full dynamics of certain variables - can be used to successfully assimilate experimental data and estimate unknown dynamics. In this example, we used a model that lacked any circadian dependencies to correctly estimate a 24-hour cycle and the mediated interaction with the SCN. Therefore our data assimilation framework can tolerate inadequate models and uncover dynamics outside the scope of the model's governing equations.

Several issues must be considered for assimilation of biological measurements. First, initial values for the filter parameters should be estimated off-line via the iterative reconstruct state/estimate parameter approach. During this off-line learning process, non-arbitrary initial values for the covariance matrices as well as model parameters can be determined. Second, we will not have access to many of the state variables for validation. Previously, we developed a system that can automatically stage the behavioral state of a freely moving animal in real time [66], based on measurements of EEG and head acceleration with a resolution of a few seconds. This process can validate the UKF's predictions of sleep-state transitions. We can also use the scored behavioral state to infer the value of the Wake-active, NREM-active, and REM-active firing rate variables. As we have shown in Fig. 7 and Fig. 8, we can then use these inferred measurements to reconstruct hidden variables and estimate unknown parameters.

It is technically feasible to measure extracellular neurotransmitter concentrations using either dialysis or electrochemical sensors. Dialysis measurements do not have the temporal resolution to resolve REM dynamics, which occur on the order of one minute or less in the rodent, or the spatial specificity to localize dynamics to sleep-wake nuclei in the rodent brain. However these measurements could be used to track and validate slow systemic dynamics such as the circadian variations that modulate the sleep-wake nuclei. In contrast, off-the-shelf electrochemical sensor technology [67] allows for highly localized measures of neurochemicals such as ACh and 5-HT with sub-second temporal resolution and sub-mm spatial resolution. Such measurements can and should be used to establish and validate models used within the data assimilation framework. In addition, they can be used to identify the subset of measurements that can be accurately reconstructed from less costly observations. An appropriate cost-function for biological data assimilation would balance the degree of reconstruction inaccuracy against the cost of obtaining risky or hard-to-access measurements.

We also note that this framework could potentially be used to choose among model dynamics. Our parameter estimation methods rely on a minimization of prediction error. A similar metric or cost function could be utilized to differentiate between UKF-based tracking and prediction of system dynamics utilizing different models with such a filter framework.

In conclusion, we have presented a data assimilation framework for combining sparse measurements together with a relatively high-dimensional nonlinear computational model to reconstruct unmeasured variables, and have demonstrated its use in the context of a model of the sleep-wake regulatory system. We have demonstrated with simulation studies that once the tracked state approaches the true system state, it reliably reconstructs the unobserved system state (Fig. 2). We have introduced a metric for ranking relative partial observability for computational models (Fig. 3) that allows us not only to assess reconstruction performance based on choice of measurement, which can serve as a guide to which system variables to measure, but also provides a methodology for optimizing filter framework parameters such as the covariance inflation (Fig. 4). In addition, we have demonstrated a parameter estimation method (Fig. 5) that allows us to track non-stationary model parameters and accommodate slow dynamics not included in the UKF model such as circadian-dependent input from the SCN (Fig. 6). Finally, we have demonstrated that we can even use observed discretized SOV, which is not one of the model variables, to successfully reconstruct model state (Figs. 7–8).

These key features will aid in the transition of this framework to the experimental bench. Our long-term plan is to develop an observer-predictor system that will track and predict sleep-wake cycles as well as the underlying state of the neural cell groups and their neurochemical environment. Because these system dynamics are implicated in and interact with numerous neurological diseases from epilepsy to schizophrenia, we anticipate that these tools will enable better understanding of the detailed interactions and provide for better, more targeted, therapies.
